# Structural, Magnetic and Luminescent Properties of Lanthanide Complexes with *N*-Salicylideneglycine

**DOI:** 10.3390/ijms16059520

**Published:** 2015-04-28

**Authors:** Ján Vančo, Zdeněk Trávníček, Ondřej Kozák, Roman Boča

**Affiliations:** 1 Regional Centre of Advanced Technologies and Materials & Department of Inorganic Chemistry, Faculty of Science, Palacký University in Olomouc, 17. listopadu 12, Olomouc CZ-77146, Czech Republic; E-Mail: jan.vanco@upol.cz; 2 Regional Centre of Advanced Technologies and Materials, Division of Metal Nanomaterials, Faculty of Science, Palacký University in Olomouc, Šlechtitelů 11, Olomouc CZ-78371, Czech Republic; E-Mail: ondrej.kozak@upol.cz; 3 Department of Chemistry, FPV, University of SS Cyril and Methodius, Trnava SK-91701, Slovakia; E-Mail: roman.boca@stuba.sk

**Keywords:** lanthanide, Schiff base, salicylideneglycine, magnetic properties, luminescent properties, X-ray structure

## Abstract

A series of anionic heavy lanthanide complexes, involving the *N*-salicylideneglycinato(2-) Schiff base ligand (salgly) and having the general formula K[Ln(salgly)_2_(H_2_O)_2_]∙H_2_O (**1**–**6**), where Ln stands for Gd, Tb, Dy, Ho, Er and Tm, was prepared using the one-pot template synthesis. The complexes were thoroughly characterized by elemental and Thermogravimetric/Differential Thermal Analyses (TG/DTA), Fourier Transform Infrared Spectroscopy (FT-IR), and photoluminescence spectroscopies, electrospray-ionization mass spectrometry, and their magnetic properties were studied by temperature-dependent dc magnetic measurements using the superconducting quantum interference device (SQUID). The X-ray structure of the terbium(III) complex (**2**), representing the unique structure between the lanthanide complexes of *N*-salicylideneamino acids, was determined. The results of spectral and structural studies revealed the isostructural nature of the prepared complexes, in which the lanthanide ion is octacoordinated by two *O*,*N*,*O*-donor salgly ligands and two aqua ligands. The analysis of magnetic data confirmed that the complexes behave as paramagnets obeying the Curie law. The results of photoluminescence spectral studies of the complexes showed the different origin in their luminescent properties between the solid state and solution. An antenna effect of the Schiff base ligand was observed in a powder form of the complex only, while it acts as a fluorophore in a solution.

## 1. Introduction

Many modern and emerging technologies, like the high-density data storage, quantum computing, or specific sensor applications [1] require new tailored and multifunctional compounds/materials for their further innovative development. Due their favourable electronic and coordination properties and the ability to act as multidentate chelate or bridging ligands, the Schiff bases represent a group of organic ligands very commonly used for the preparation of coordination compounds showing interesting physical (e.g., spin crossover properties, single molecule/chain magnetism, luminescence, non-linear optic properties, *etc.*) or chemical properties (e.g., favourable redox properties, selective reactivity towards specific molecules, *etc.*), as well as biological activities (e.g., antimicrobial, antiradical, radioprotective, antidiabetic, anticancer, *etc.*) [2,3,4,5,6,7,8,9,10]. A very abundant subgroup of Schiff base metal complexes, which can meet the requirements for the multifunctional materials, is represented by the rare earth metal complexes, which possess the interesting luminescent properties [11,12,13], magnetic properties (e.g., single molecule, or single chain magnetism) [14,15] and promising biological properties (e.g., antimicrobial, or anticancer) [11,12,13]. On the other hand, the lanthanide complexes, involving the Schiff bases formed by the condensation of aromatic *o*-hydroxy-aldehydes and amino acids, are not so common. To date, a series of lanthanide complexes with Schiff bases, prepared by the condensation of salicylaldehyde and naphthaldehyde derivatives (such as *o*-vanilline, or 5-bromo-2-hydroxybenzaldehyde) with lysine [16], 6-aminolysine [16], phenylalanine [17], tyrosine [18], glutamic acid [18], aspartic acid [19], valine [18,20], leucine [21], glutamine [21], alanine [21], and glycine [21,22,23,24], has been reported. Due to coordination variability of the Schiff base ligands and the coordination properties of the lanthanide central atoms (ability to employ the coordination numbers up to 12), the structures of these complexes are quite divergent. When we narrow our focus only on the group of the lanthanide complexes of *N*-salicylideneglycine (H_2_L), electroneutral aqua-complexes with the general formula Ln(L)(HL)∙*x*H_2_O, where Ln = La, Ce, Pr, Nd, Sm, Eu, and *x* = 3–3.5 [22], complexes of the composition [Ln(HL)_2_(Y)(H_2_O)_0-1_] [23], and ternary complexes with the general formula [Ln(L)(bpy)(Y)]∙H_2_O [24], where Ln = lanthanide, and Y = ${\text{NO}}_{3}^{-}$, Cl^−^, have been prepared and characterized up to now. However, there are no reports on lanthanate anionic complexes containing the *N*-salicylideneglycine ligand in the literature. Therefore, we decided to investigate these compounds, and we prepared and characterized a series of anionic complexes having the general formula K[Ln(salgly)_2_(H_2_O)_2_]∙H_2_O, where Ln represents one of the heavy lanthanide metals selected from the group Gd, Tb, Dy, Ho, Er, and Tm, and to study their structural, magnetic and photoluminescent properties with the aim to find any applicable feature of them.

## 2. Results and Discussion

### 2.1. Synthesis of Complexes

**Scheme 1:**
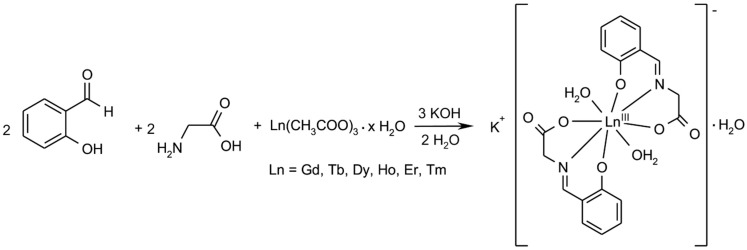
Schematic representation of the reaction pathway leading to complexes **1**–**6**.

The lanthanide complexes of *N*-salicylideneglycine (**1**–**6**) were prepared by the one-pot template synthesis from the reaction mixture, containing one molar equivalent of lanthanide acetate (Ln(ac)_3_·*x*H_2_O, where Ln = Gd, Tb, Dy, Ho, Er, Tm, and *x* = 0–6), three molar equivalents of potassium hydroxide, two molar equivalents of 2-hydroxybenzaldehyde (salicylaldehyde) and two molar equivalents of glycine (Gly) (Scheme 1). Concretely, the equal volumes of the water solution of lanthanide acetate, glycine, and potassium hydroxide (10 mmol of Ln(ac)_3_·*x*H_2_O + 20 mmol Gly + 30 mmol KOH in 25 mL) and salicylaldehyde solution in 96% ethanol (20 mmol in 25 mL) were mixed together and stirred at 60 °C for 1 h. Crystals, suitable for X-ray structure analysis in the case of complex **2**, were formed by the slow cooling of the dark yellow solutions to laboratory temperature overnight.

### 2.2. Characterization of Compounds

Crystals of complexes **1**–**6**, formed by the cooling and slow evaporation of the reaction mixture, were filtered off, washed with 96% ethanol (2 × 5 mL) and dried in a desiccator over KOH. The final products were characterized by elemental analysis, Thermogravimetric/Differential Thermal Analysis (TG/DTA), Fourier Transform Infrared Spectroscopy (FT-IR) and photoluminescence spectroscopy, mass spectrometry, dc magnetic measurements using the superconducting quantum interference device (SQUID), and by single crystal X-ray structural analysis in the case of the terbium complex **2**.

Characterization of complex **1**: Potassium [diaqua-bis(*N*-salicylideneglycinato)gadolinate(III)] monohydrate.

Yellowish microcrystals (yield η = 83%).

Elemental analysis (Calculated/Found) for C_18_H_20_N_2_O_9_KGd (*M*_r_ = 604.707): C, 35.75; H, 3.33; N, 4.63. Found: C, 35.76; H, 3.40; N, 4.55%.

FT-IR (ATR, cm^−1^), signal intensities are defined as w = weak, m = medium, and s = strong: 3338m ν(O–H), 3048s ν(C–H)_arom_, 2905m ν(C–H)_aliphatic_, 1627s ν(C=N), 1559s, 1543s ν_asym_(COO), 1469m ν(C=C)_arom_, 1447m ν_sym_(COO), 1301m ν(C–O)_arom_, 1068w δ(C–H)_arom_, 756m δ(H–C–H).

Far-IR (Nujol, cm^−1^), signal intensities are defined as w = weak, m = medium, and **s** = strong: 461w ν(Gd–O), and 411m ν(Gd–N).

Electrospray-ionization mass spectrometry measured in methanol solutions (*m*/*z*; [the corresponding species]^−^): 512.15 [Gd(salgly)_2_]^−^, 530.18 [Gd(salgly)_2_(H_2_O)]^−^, 577.11 [Gd(salgly)_2_(H_2_O)(OH)+K]^−^, 642.15 [Gd(salgly)_2_(H_2_O)(OH)+K+2CH_3_OH]^−^.

Characterization of complex **2**: Potassium [diaqua-bis(*N*-salicylideneglycinato)terbiate(III)] monohydrate.

Yellowish microcrystals (yield η = 92%).

Elemental analysis (Calcd./Found) for C_18_H_20_N_2_O_9_KTb (*M*_r_ = 606.382): C, 35.65; H, 3.32; N, 4.62. Found: C, 35.75; H, 3.18; N, 4.42%.

FT-IR (ATR, cm^−1^), signal intensities are defined as w = weak, m = medium, and s = strong: 3333m ν(O–H), 3048s ν(C–H)_arom_, 2902m ν(C–H)_aliphatic_, 1627s ν(C=N), 1558s, 1542s ν_asym_(COO), 1470m ν(C=C)_arom_, 1447m ν_sym_(COO), 1300m ν(C–O)_arom_, 1066w δ(C–H)_arom_, 755m δ(H–C–H).

Far-IR (Nujol, cm^−1^), signal intensities are defined as w = weak, m = medium, and s = strong: 460w ν(Tb–O), and 411m ν(Tb–N).

Electrospray-ionization mass spectrometry measured in methanol solutions (*m*/*z*; [the corresponding species]^−^): 513.10 [Tb(salgly)_2_]^−^, 531.00 [Tb(salgly)_2_(H_2_O)]^−^, 561.87 [Tb(salgly)_2_(OH)+K]^−^, 610.81 [Tb(salgly)_2_(H_2_O)(OH)+K+CH_3_OH]^−^.

Characterization of complex **3**: Potassium [diaqua-bis(*N*-salicylideneglycinato)dysprosiate(III)] monohydrate.

Yellow microcrystals (yield η = 82%).

Elemental analysis (Calcd./Found) for C_18_H_20_N_2_O_9_KDy (*M*_r_ = 609.957): C, 35.44; H, 3.30; N, 4.59. Found: C, 35.20; H, 3.50; N, 4.61%.

FT-IR (ATR, cm^−1^), signal intensities are defined as w = weak, m = medium, and s = strong: 3330m ν(O–H), 3049s ν(C–H)_arom_, 2900m ν(C–H)_aliphatic_, 1628s ν(C=N), 1557s, 1542s ν_asym_(COO), 1470m ν(C=C)_arom_, 1445m ν_sym_(COO), 1300m ν(C–O)_arom_, 1067w δ(C–H)_arom_, 755m δ(H–C–H).

Far-IR (Nujol, cm^−1^), signal intensities are defined as w = weak, m = medium, and s = strong: 461w ν(Dy–O), and 413m ν(Dy–N).

Electrospray-ionization mass spectrometry measured in methanol solutions (*m*/*z*; [the corresponding species]^−^): 518.13 [Dy(salgly)_2_]^−^, 536.14 [Dy(salgly)_2_(H_2_O)]^−^, 575.10 [Dy(salgly)_2_(H_2_O)(OH)+Na]^−^, 583.11 [Dy(salgly)_2_(H_2_O)(OH)+K]^−^.

Characterization of complex **4**: Potassium [diaqua-bis(*N*-salicylideneglycinato)holmiate(III)] monohydrate.

Yellowish microcrystals on sunlight, Pale orange microcrystals under the fluorescence light (yield η = 80%).

Elemental analysis (Calcd./Found) for C_18_H_20_N_2_O_9_KHo (*M*_r_ = 612.387): C, 35.30; H, 3.29; N, 4.57. Found: C, 35.17; H, 3.18; N, 4.20%.

FT-IR (ATR, cm^−^^1^), signal intensities are defined as w = weak, m = medium, and s = strong: 3334m ν(O–H), 3048s ν(C–H)_arom_, 2905m ν(C–H)_aliphatic_, 1629s ν(C=N), 1565s, 1544s ν_asym_(COO), 1469m ν(C=C)_arom_, 1447m ν_sym_(COO), 1300m ν(C–O)_arom_, 1067w δ(C–H)_arom_, 756m δ(H–C–H).

Far-IR (Nujol, cm^−1^), signal intensities are defined as w = weak, m = medium, and s = strong: 461w ν(Ho–O), and 414m ν(Ho–N).

Electrospray-ionization mass spectrometry measured in methanol solutions (*m*/*z*; [the corresponding species]^−^): 519.13 [Ho(salgly)_2_]^−^, 537.13 [Ho(salgly)_2_(H_2_O)]^−^, 576.06 [Ho(salgly)_2_(H_2_O)(OH)+Na]^−^, 584.12 [Ho(salgly)_2_(H_2_O)(OH)+K]^−^.

Characterization of complex **5**: Potassium [diaqua-bis(*N*-salicylideneglycinato)erbiate(III)] monohydrate.

Yellow microcrystals (yield η = 90%).

Elemental analysis (Calcd./Found) for C_18_H_20_N_2_O_9_KEr (*M*_r_ = 614.716): C, 35.17; H, 3.28; N, 4.56. Found: C, 35.01; H, 3.23; N, 4.27%.

FT-IR (ATR, cm^−1^), signal intensities are defined as w = weak, m = medium, and s = strong: 3338m ν(O–H), 3048s ν(C–H)_arom_, 2905m ν(C–H)_aliphatic_, 1629s ν(C=N), 1560s, 1542s ν_asym_(COO), 1470m ν(C=C)_arom_, 1449m ν_sym_(COO), 1301m ν(C–O)_arom_, 1068w δ(C–H)_arom_, 756m δ(H–C–H).

Far-IR (Nujol, cm^−1^), signal intensities are defined as w = weak, m = medium, and s = strong: 460w ν(Er–O), and 415m ν(Er–N).

Electrospray-ionization mass spectrometry measured in methanol solutions (*m*/*z*; [the corresponding species]^−^): 520.13 [Er(salgly)_2_]^−^, 538.01 [Er(salgly)_2_(H_2_O)]^−^, 578.14 [Er(salgly)_2_(H_2_O)(OH)+Na]^−^, 585.14 [Er(salgly)_2_(H_2_O)(OH)+K]^−^.

Characterization of complex **6**: Potassium [diaqua-bis(*N*-salicylideneglycinato)tuliate(III)] monohydrate.

Yellow microcrystals (yield η = 78%).

Elemental analysis (Calcd./Found) for C_18_H_20_N_2_O_9_KTm (*M*_r_ = 616.391): C, 35.07; H, 3.27; N, 4.54. Found: C, 34.83; H, 3.14; N, 4.25%.

FT-IR (ATR, cm^−1^), signal intensities are defined as w = weak, m = medium, and s = strong: 3331m ν(O–H), 3049s ν(C–H)_arom_, 2905m ν(C–H)_aliphatic_, 1629s ν(C=N), 1561s, 1542s ν_asym_(COO), 1471m ν(C=C)_arom_, 1448m ν_sym_(COO), 1300m ν(C–O)_arom_, 1068w δ(C–H)_arom_, 755m δ(H–C–H).

Far-IR (Nujol, cm^−1^), signal intensities are defined as w = weak, m = medium, and s = strong: 461w ν(Tm–O), and 417m ν(Tm–N).

Electrospray-ionization mass spectrometry measured in methanol solutions (*m*/*z*; [the corresponding species]^−^): 523.13 [Tm(salgly)_2_]^−^, 541.13 [Tm(salgly)_2_(H_2_O)]^−^, 588.07 [Tm(salgly)_2_(H_2_O)(OH)+K]^−^.

#### 2.2.1. X-ray Structure of Complex **2**

**Figure 1:**
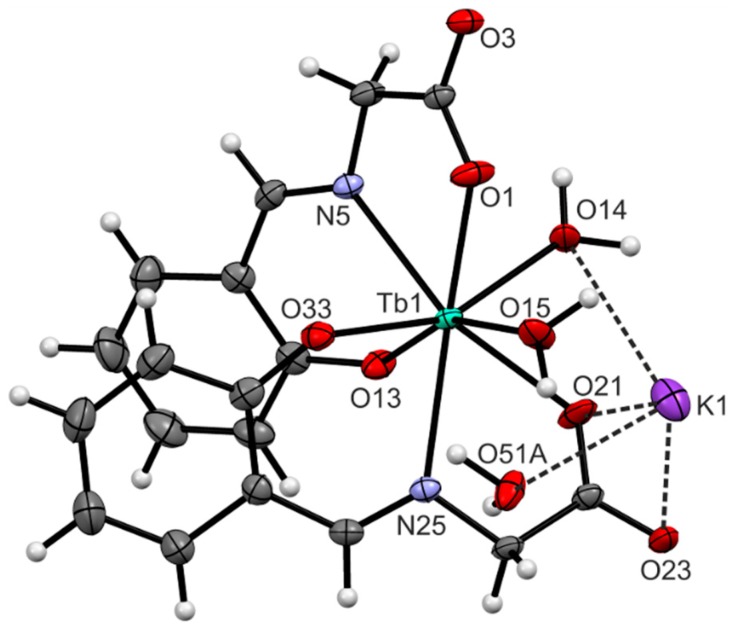
The crystallographically independent part of complex **2**. The O51A atom is disordered over two positions. The position with the higher occupancy factor (0.61) is displayed only owing to clarity.

**Figure 2:**
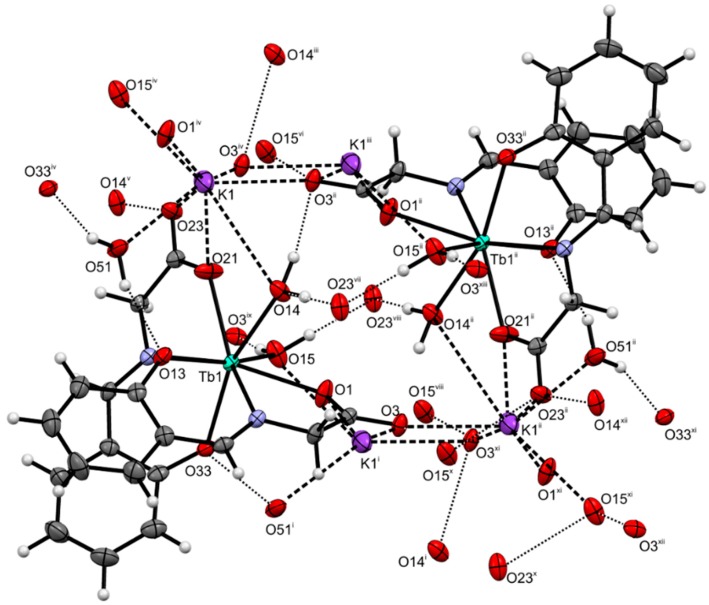
A part of crystal structure of complex **2**, showing variety of O–H···O hydrogen bonds (dotted lines) and electrostatic non-bonding K···O interactions (dashed lines). (Symmetry codes: (i) *x*, 1 + *y*, *z*; (ii) 1/2 − *x*, 3/2 − *y*, 1 − *z*; (iii) 1/2 − *x*, 1/2 − *y*, 1 − *z*; (iv) *x*, −1 + *y*, *z*; (v) *x*, 1 − *y*, 1/2 + *z*; (vi) 1/2 − *x*, −1/2 + *y*, 3/2 − *z*; (vii) *x*, 1 − *y*, −1/2 + *z*; (viii) 1/2 − *x*, 1/2 + *y*, 3/2 − *z*; (ix) *x*, 2 − *y*, 1/2 + *z*; (x) *x*, 2 − *y*, −1/2 + *z*; (xi) 1/2 − *x*, 2.5 − *y*, 1 − *z*; (xii) 1/2 − *x*, 1/2 + *y*, 1/2 − *z*; (xiii) 1/2 − *x*, −1/2 + *y*, 1/2 − *z*).

**Figure 3:**
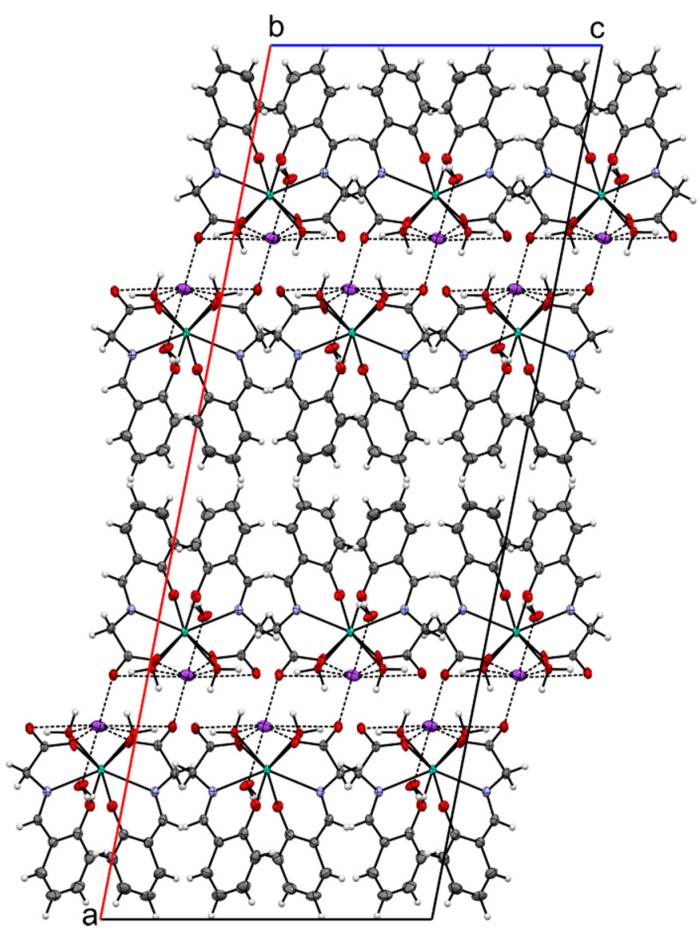
A part of the crystal structure of complex **2** (view along the *b*-axis), showing formation of 2D supramolecular layers.

The crystallographically independent part of the unit cell of K[Tb(salgly)_2_(H_2_O)_2_]∙H_2_O (**2**) is depicted in Figure 1. The crystal data and structure refinement are presented in Table 1. The selected bond lengths and angles are listed in Table 2. The terbium(III) atom is octacoordinated by two salgly and two aqua ligands with an N_2_O_6_ donor set. The coordination polyhedron can be described as biaugmented trigonal prism J50 (as determined by the best similarity parameter in SHAPE 2.1. software [25]). The potassium counter-ion is electrostatically octacoordinated with the oxygen atoms of the carboxyl groups from the Schiff base ligands, with terbium-coordinated water molecules and crystal water molecules, with the K···O distances being 2.659(6)–3.288(2) Å, and with the O51 atoms disordered over two positions. Furthermore, the crystal structure of complex **2** is stabilized by an extensive network of O–H···O hydrogen bonds and K···O non-covalent contacts (see Table 3, Figure 2 and Figure 3), thus forming a 2D-layered supramolecular structure.

**Table 1:** Crystal data and structure refinement for complex **2**.

Empirical Formula	C_18_H_20_KN_2_O_9_Tb
Formula weight	606.38
Temperature	120(2) K
Wavelength	0.71075 Å
Crystal system, space group	monoclinic, *C*2*/c*
Unit cell dimensions	*a* = 38.255(7) Å
	*b* = 8.0722(6) Å
	*c* = 14.2464(14) Å
	α = γ = 90° β = 101.030(13)°
Volume	4318.0(1) Å^3^
*Z*, Calculated density	8, 1.866 g·cm^−3^
Absorption coefficient	3.520 mm^−1^
*F*(000)	2384
Crystal size	0.04 × 0.04 × 0.01 mm
*θ* range for data collection	2.58° to 25.00°
Limiting indices	−45 ≤ *h* ≤ 44, −9 ≤ *k* ≤ 9, −15 ≤ *l* ≤ 16
Reflections collected/unique	13,280/3782, [*R*(int) = 0.0141]
Completeness to θ = 25°	99.2%
Absorption correction	Semi-empirical from equivalents
Max. and min. transmission	1.000 and 0.368
Refinement method	Full-matrix least-squares on *F*^2^
Data/restraints/parameters	3782/16/314
Goodness-of-fit on F^2^	0.994
Final *R* indices [*I* > 2σ(*I*)]	*R*1 = 0.0186, w*R*2 = 0.0498
*R* indices (all data)	*R*1 = 0.0192, w*R*2 = 0.0501
Largest differences in peak and hole	0.377 and −0.530 e. Å^−3^

**Table 2:** Selected interatomic parameters [Å, °] for complex **2**.

Distance	[Å]	Angle	[°]
Tb1–O1	2.398(2)	O1–Tb1–N25	141.43(6)
Tb1–N5	2.551(2)	O21–Tb1–N5	142.21(7)
Tb1–O13	2.230(2)	O33–Tb1–O14	157.26(6)
Tb1–O21	2.396(2)	O15–Tb1–O13	156.98(6)
Tb1–O14	2.462(2)	O15–Tb1–N5	130.21(7)
Tb1–O15	2.458(2)	O1–Tb1–O13	135.78(6)
Tb1–O33	2.246(2)	O1–Tb1–O15	67.24(6)
Tb1–N25	2.552(2)	O14–Tb1–O15	99.61(6)
K1···O14	3.254(2)	O21–Tb1–O15	76.28(7)
K1···O21	2.775(2)	N25–Tb1–O15	79.33(6)
K1···O23	3.288(2)	O33–Tb1–O13	92.29(7)
K1···O51A ^a^	2.659(6)	N25–Tb1–O13	79.00(6)
K1···O51B ^a^	2.704(4)	O33–Tb1–O15	88.02(7)

^a^ The disordered atoms of O51.

**Table 3:** Hydrogen bond geometry (Å, °) in the crystal structure of complex **2**.

D–H···A	*d*(D–H)	*d*(H···A)	*d*(D···A)	<(DHA)
O14–H14A···O23 ^vii^	0.919(14)	1.768(14)	2.673(2)	168(3)
O14–H14B···O1 ^ii^	0.945(14)	2.60(3)	3.283(3)	129(2)
O14–H14B···O3 ^ii^	0.945(14)	2.030(14)	2.968(3)	172(3)
O15–H15A···O3 ^ix^	0.927(14)	1.760(14)	2.683(2)	173(3)
O15–H15B···O23 ^viii^	0.923(14)	1.790(14)	2.712(2)	177(3)
O51A ^a^–H51A···O13	0.960(18)	1.965(18)	2.882(5)	159(5)
O51A ^a^–H51B···O33 ^iv^	0.962(19)	1.870(19)	2.768(5)	154(5)
O51B ^a^–H51C···O13	0.937(17)	1.935(17)	2.805(4)	153(4)
O51A ^a^–H51D···O33 ^iv^	0.988(16)	1.907(16)	2.864(4)	162(4)

(Symmetry codes: (ii) 1/2 − *x*, 3/2 − *y*, 1 − *z*; (iv) *x*, − 1 + *y*, *z*; (vii) *x*, 1 − *y*, −1/2 + *z*; (viii) 1/2 − *x*, 1/2 + *y*, 3/2 − *z*; (ix) *x*, 2 − *y*, 1/2 + *z*). ^a^ The disordered atoms of O51.

To the best of our knowledge, there are only three structures of lanthanide complexes involving the Schiff bases derived from aromatic 2-hydroxy-aldehydes and amino acids, and deposited within the Cambridge Structural Database (CSD, version 5.35, update May 2014) [26]. The first two are enantiomeric complexes of trisodium bis(2-oxido-3-methoxybezylidene-(*R*/*S*)-aspartato)europiate(III) tetrahydrate [19], in which the Schiff base differs in the denticity; however, the coordination polyhedron of the europium(III) is very similar to that in complex **2** and adopts the shape of highly regular tetragonal antiprisms. Moreover, the sodium counterions interact electrostatically at the same places as in complex **2**, *i.e*., with the oxygen atoms of carboxylate groups and water molecules forming a 2D-supramolecular network of electrostatic and non-bonding interactions leading to the formation of layered structure. The third structure is much more complicated, as it involves a heptanuclear dysprosium(III) complex of the Schiff base formed by the condensation of 2-hydroxy-naphthaldehyde and 2-aminoisobutyric acid [27]. It forms the irregular [Ln_7_] clusters with the combination of different coordination polyhedra, and, thus, it cannot be used as a suitable reference.

#### 2.2.2. Spectral Analyses

**Figure 4:**
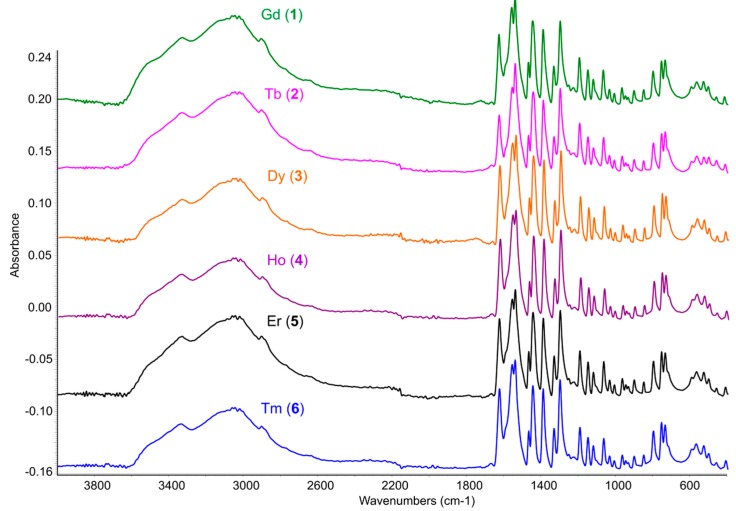
The overview of FT-IR spectra of K[Ln(salgly)_2_(H_2_O)_2_]·H_2_O (**1**–**6**), where Ln represents Gd, Tb, Dy, Ho, Er and Tm.

The overview of FT-IR spectra of the prepared lanthanide complexes **1**–**6** is shown in Figure 4.

A first glimpse at these spectra indicates their similarity, which may be associated with the assumption that the prepared complexes are most likely isostructural. This assumption may also be supported by comparing the powder X-ray diffraction patterns of complexes **1**–**6**, which contained the similar diffraction positions (see Figure S1 in the Supplementary Information). A broad band in the spectral region of 3338–3330 cm^−1^, which corresponds to the O–H stretching vibration, can be connected with the presence of water molecules within the structures of the complexes. The bands centred at circa 3049 cm^−1^ may be associated with the stretching vibrations of the aromatic C–H bonds, while the bands observed at about 2900 cm^−1^ can be attributed to the symmetric and asymmetric vibrations of the CH_2_ group. Other important bands detected between 1627 and 1629 cm^−1^ can be associated with the C=N stretching vibrations of the azomethine group of the Schiff base. The peaks observed at 1557–1565 cm^−1^ and 1542–1544 cm^−1^ can belong to the asymmetric vibrations of the carboxyl group, indicating probably the small difference in the mode of coordination between the two types of monodentately-coordinated carboxyl groups in the structures of the complexes. The skeletal vibrations of the aromatic C–C bonds appeared at 1469–1471 cm^−1^, while the symmetric vibrations of the carboxyl groups were observed in the region of 1445–1449 cm^−1^. The C_ar_–O stretching vibrations of the phenolate moiety were observed at 1300–1301 cm^−1^, and the less intensive out-of-plane deformation vibrations of aromatic C–H bonds and H–C–H group vibrations were detected at 1066–1068, and 755–756 cm^−1^, respectively. These findings are in good agreement with our previously published transition metal complexes, involving the similar Schiff base derived from β-alanine [28]. In the far-IR region, two vibrations 460–461 and 411–415 cm^−1^ were identified, assignable to the Ln–O, and Ln–N stretching vibrations, respectively, which is also in agreement with the literature data [29].

**Figure 5:**
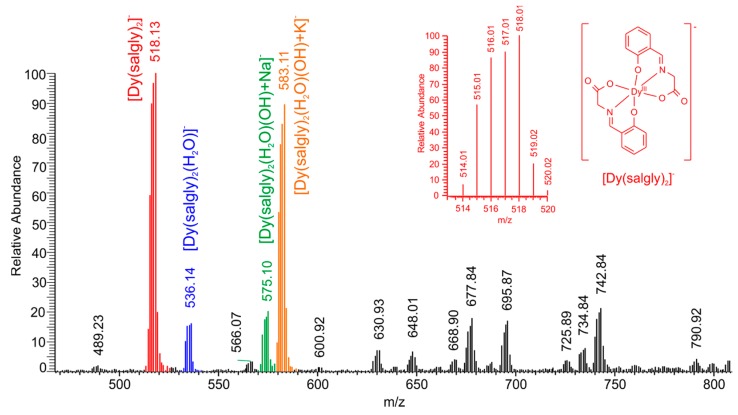
The example of the electrospray-ionization mass spectrum of K[Dy(salgly)_2_(H_2_O)_2_]·H_2_O (**3**). Inset represents the ideal isotopic distribution corresponding to the species shown.

The analysis of electrospray-ionization mass spectra, measured in the negative ionization mode (a representative example of the mass spectra of the dysprosium complex **3** is presented in Figure 5), confirmed the presence of peaks assignable, according to the mass (*m*/*z*) and isotopic distribution, to the molecular anionic species [Ln(salgly)_2_]^−^ in all the measured spectra, accompanied by a rich variety of pseudomolecular species derived from these anions, such as [Ln(salgly)_2_(H_2_O)]^−^, [Ln(salgly)_2_(H_2_O)(OH)+Na]^−^, [Ln(salgly)_2_(H_2_O)(OH)+K]^−^, and [Ln(salgly)_2_(H_2_O)(OH)+K+*x*CH_3_OH]^−^, where *x* = 1–2, respectively.

**Figure 6:**
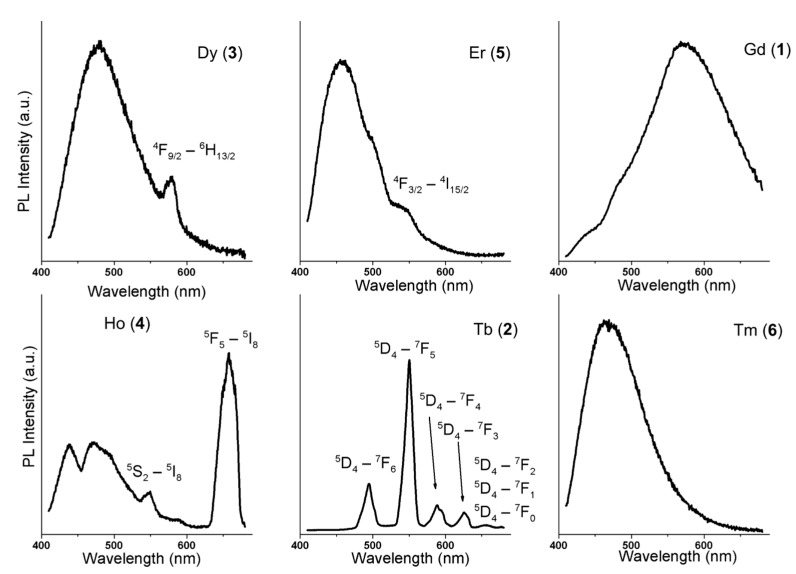
The emission spectra of the complexes **1**–**6**, λ_ex_ = 350 nm.

The photoluminesce spectra of the studied lanthanide complexes in solid state and in water solutions were measured in the visible spectral region under ultraviolet A (UVA) light excitation (λ_ex_ = 350 nm). The background-corrected emission photoluminescence spectra of the complexes **1**–**6** are shown in Figure 6.

Three types of emission spectra were identified during the luminescence measurements:

(a) the spectrum showing a broad peak (complex **1**, complex **6**);
(b) the spectrum showing several narrow peaks (complex **2**);
(c) the spectrum showing a combination of both broad and narrow peaks (complexes **3**–**5**).

Generally, it is well known that lanthanide-containing compounds are luminescent due to *f*–*f* electronic transitions exhibiting sharp emission lines in the UV, Visible and Near-infrared (NIR) spectral regions [30]. Such *f*–*f* transitions-related bands were identified in the spectra of complexes **2**, **3**, **4**, and **5** and are shown in Figure 6. Typically, broad emission bands are not observed for Ln^3+^ ions due to the absence of molecular/lattice vibrations, which typically cause the homogeneous spectral broadening. Moreover, the 4*f*-electrons, which play a role in optical transitions, do not participate much in binding (the covalency of an Ln^3+^–ligand bond is at most 5%–7%) so the excitation-induced rearrangement does not affect the binding pattern in the molecules. Therefore, the internuclear distances remain almost unchanged after the excitation, which generates small Stokes shifts. On the other hand, in organic compounds, the electronic transitions are considerably affected by molecular vibrations, and the excitation often leads to the lengthening of chemical bonds. Thus, the emission bands are usually much broader with larger Stokes shifts [30].

**Figure 7:**
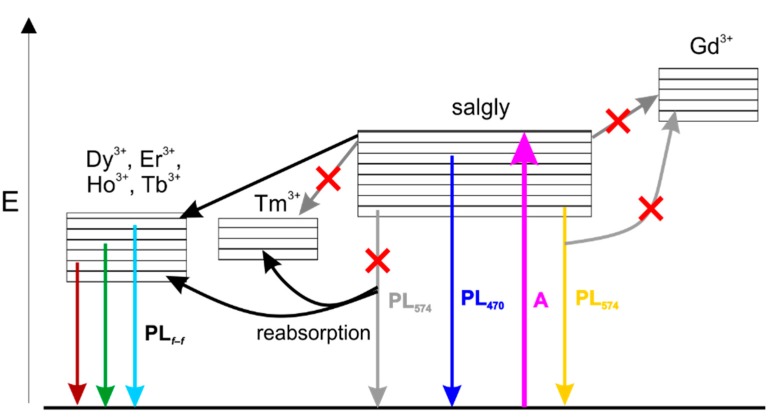
Schematic representation of the energy transfer processes taking place in the studied lanthanide complexes (powders): Energy absorption (A), emission (PL) and intersystem crossings. Red croses mean improbable processes.

Therefore, the broad band observed at 460–480 nm in all the complexes, except for complex **2**, can be assigned to emissions arising from the ligand since it incorporates suitable conjugated system. In complex **1**, another broad and more intense band centred at 574 nm was observed. Due to the isostructurality of the complexes (*vide supra*), the reason for different spectral shapes probably lies in the distinctively different properties of the central lanthanide ion. Indeed, the lowest excited state of Gd^3+^, however, is too high to receive energy from most organic ligands [31] and thus complex **1** shows the photoluminescence of the ligand in the absence of charge transfer to the central atom. Contrary to that, the central metal ions in complexes **3**–**6** posses energy levels capable of absorbing the energy of the transition that is manifested as emission at 574 nm (observed for complex **1**) [30]. In complex **2**, no broad band is apparent, perhaps due to masking it by a very strong emission originating in *f*–*f* transitions in Tb^3+^ central ions. The luminescence intensity of complex **2** was found roughly two orders of magnitude higher than that of the other complexes measured at similar conditions and thus the broadband emission would hardly be observable, if present. On the other hand, low efficiency of the ligand-to-metal energy transfer probably causes no sharp emissions from *f*–*f* transitions in the case of complex **6**. The considered energy transfers between the Schiff base ligand and lanthanide ions as well as the optical transitions are schematically depicted in Figure 7.

**Figure 8:**
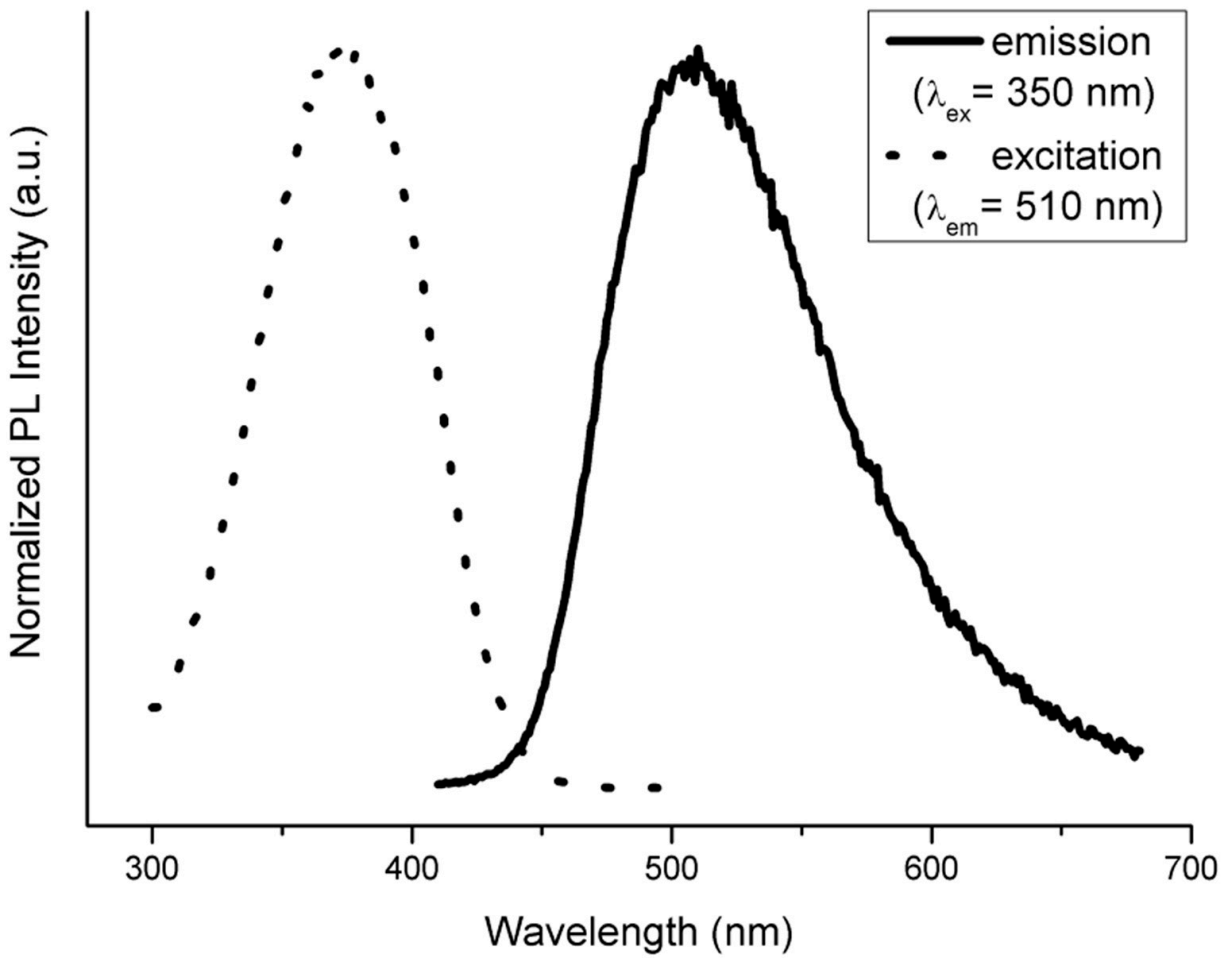
The excitation and emission spectra of water solution of potassium salt of *N*-salicylideneglycinate. The dotted line represents the excitation spectrum and the solid line represents the region of emission maxima at 510 nm.

The proof that the salgly ligand is fluorescent itself in aqueous solution (as shown in Figure 8) was obtained by measuring the solution of its potassium salt.

**Figure 9:**
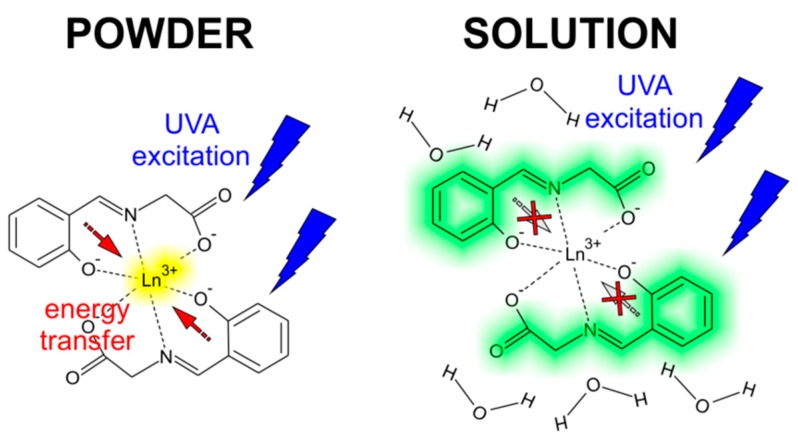
The proposed routes of the energy transfer influencing the photoluminescence of the complexes in the emission spectra measured in the solid state (**left**) and in water solutions (**right**).

All the prepared complexes **1**–**6** were also measured in aqueous solution and, interestingly, they exhibit identical spectral characteristics as those of the pure ligand. It is obvious, that the central lanthanide atom in these complexes makes no difference to the photoluminescence spectra shape when dissolved and the luminescence thus has its origin in the aromatic system of the ligand in aqueous solution. In the previous paragraphs, we assigned the broad band observed in the powdered samples at approximately 460–480 nm (see Figure 6) to the aromatic ligand as well. The solutions of the complexes, however, show this emission red shifted to ~510 nm, most likely due to the solvent relaxation. The reason for not observing any sharp *f*–*f* transition-related features from dissolved complexes probably relates to the effect of solvent on the luminescence of the ligand. In the absence of the solvent, it primarily harvests the energy for subsequent metal ion excitation via energy transfer. The presence of the broad, ligand-assigned photoluminescence in powdered samples should be caused by the low efficiency of the energy transfer [32]. However, when the water molecules solvate the complex, the energy transfer is blocked almost completely and the observed luminescence arises entirely from the aromatic system of the Schiff base ligand. We propose a plausible explanation for this observation based on dynamics of the system. Considering the typically very fast time scale of solvent relaxation in water (<50 fs) [33] and the principally much longer process that leads to ligand-to-metal energy transfer [32], it can be expected that the relaxed excited emissive state of the ligand is reached prior to energy transfer may occur. Depending on the phase of the sample (*i.e.*, the solid state *vs.* solution), the ligand may act as an antenna (in powder) or a fluorophore (in solution) as depicted in Figure 9.

#### 2.2.3. Thermal Analysis

**Figure 10:**
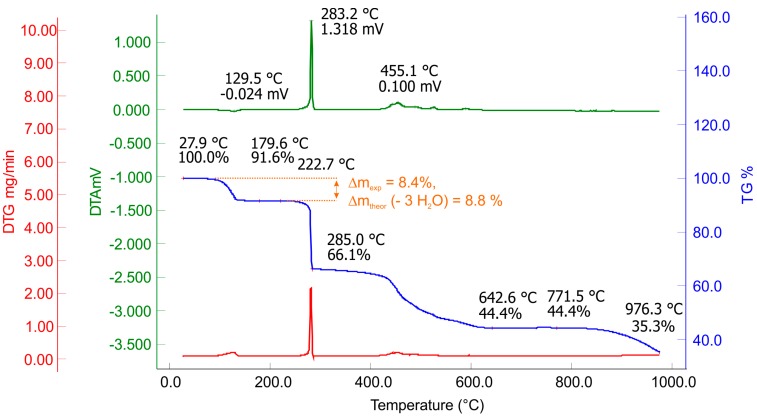
DTA/TG/DTG curves for complex **4**.

The simultaneous TG and DTA analyses were performed for complex **4** as a representative sample (see Figure 10). The complex starts to decompose at 80 °C and the first decomposition step is finished at *ca*. 180 °C, and accompanied by a small *endo*-effect on DTA curve with the maximum centred at 130 °C. This weight loss may be associated with the elimination of one crystal water molecule and two aqua ligands (Δ*m* = 8.4/8.8% found/calcd.). The dehydrated complex of the composition K[Ho(salgly)_2_] is thermally stable up to *ca.* 220 °C and then a sharp weight loss accompanied by an *exo*-effect with the maximum centred at *ca.* 280 °C, associated with the decomposition of the complex connected with the Schiff base ligand’s oxidation, is apparent from TG/DTA curves. Further, the complex intermediate is decomposed in two waves and the thermal decay is not finished even at 976 °C. A plateau occurs between 643 and 772 °C, which may be connected with the formation of holmium(III) carbonate, Ho_2_(CO_3_)_3_, containing a small amount of an unidentified impurity (Δ*m* = 44.4/41.6% found/calcd.), which starts to decompose to a mixture of holmium oxides at *ca.* 820 °C.

#### 2.2.4. Magnetic Measurements

The solid state temperature dependence of magnetic susceptibility was measured over the temperature range of 300–1.8 K for all the prepared complexes (**1**–**6**) in the quest to describe their magnetic properties. As it turned out, this task is very complicated due to the effects of magnetic anisotropy found in the Ln^3+^ ions with unevenly filled 4*f*-orbitals and the effects of the crystal field of the ligands. Therefore, we decided to present here the results obtained by fitting the experimental data into an idealized model based on the Curie law with an added molecular field term using Hamiltonian in the form $\hat{H}=g_{iso}\mu_{B}B\hbar^{-1}{\hat{S}}_{Z}\;$ and the explicit formulae (1) for the fitting of temperature-dependent data. In all the cases, the complexes behaved as paramagnets and followed the Curie law in the wide range of temperatures.

(1) $$\text{χ}=\frac{{\text{χ}}_{0}}{1-\left[\left(zj/k\right)/\left(C_{0}g_{iso}^{2}\right)\right]\times {\text{χ}}_{0}}+\text{α}$$

where ${\text{χ}}_{0}=C_{0}g_{iso}^{2}\times \frac{J\left(J+1\right)/3}{T}$, and $C_{0}=N_{A}{\text{μ}}_{0}{\text{μ}}_{B}^{2}/k$.

**Figure 11:**
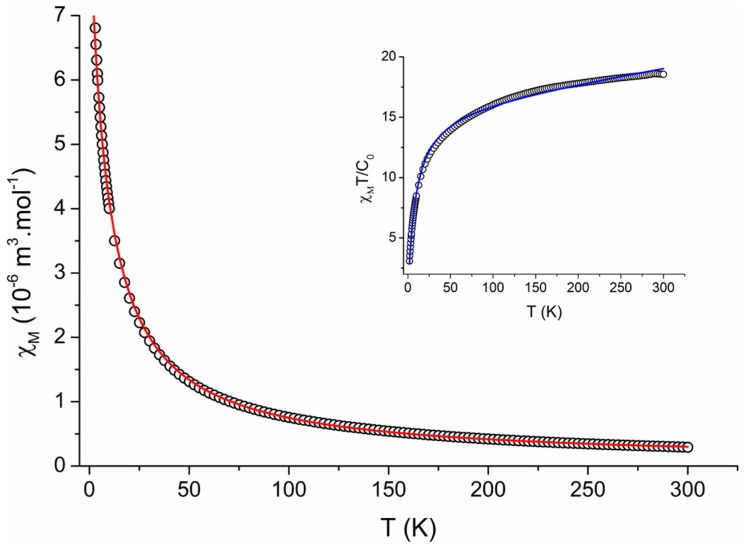
The temperature-dependence of the magnetic susceptibility (χ_M_, ○) and χ_M_T/C_0_ (inset) for complex **6**. The best fit of χ_M_ is represented by the solid line.

The best fitting parameters for the complexes **1**–**6** are presented in Table 4 and an example of fitting of the temperature-dependence of susceptibility data for complex **6** is presented in Figure 11. The obtained values correspond well with those reported in literature [34].

**Table 4:** Best fitting parameters of temperature dependence of magnetic susceptibility for complexes (**1**–**6**).

Complex	Angular Momentum (*J*)	*g*(theor) [34]	Landé *g*-Factor (*g*_iso_)	Curie Constant (*C*, m^3^∙mol^−1^)	Molecular Field Term (*zj·*cm^−1^)	α_TIM_ (10^−9^ m^3^∙mol^−1^)	Fit Error (%) *
**1**	3.5	2	2.0259	1.0158 × 10^−4^	0.01164	−0.08274	0.42
**2**	6.0	3/2	1.4154	1.3222 × 10^−4^	−0.13160	65.64282	6.12
**3**	7.5	4/3	1.3483	1.8211 × 10^−4^	−0.03634	−18.40887	6.46
**4**	8.0	5/4	1.2614	1.8002 × 10^−4^	−0.23447	−0.21585	7.91
**5**	7.5	6/5	1.1001	1.2121 × 10^−4^	−0.05287	135.79771	8.80
**6**	6.0	7/6	1.0322	7.0312 × 10^−5^	−0.38454	87.07327	1.97

* The fit error is defined as the relative error of the χ_M_ fitting by the relation:
$$\text{σ}\left(\text{χ}\right)=100\times \overset{N}{\underset{n=1}{\sum }}\left({\text{χ}}_{n}^{exp}-{\text{χ}}_{n}^{calc}\right)/{\text{χ}}_{n}^{exp}$$

Theory of the magnetism of 4*f*-multiplets predicts [35] that for the electron configurations *f^n^* with *n* > 7 the ground state is well separated from the excited ones so that the Curie law is perfectly obeyed for $g_{J\neq 0}=1+\frac{\left(g_{e}-1\right)\left[\;J\left(\;J+1\right)-L\left(L+1\right)+S\left(S+1\right)\right]}{\left[2J\left(\;J+1\right)\right]}$. The measured susceptibility data confirms that this is well fulfilled for the high-temperature region of the magnetic susceptibility, at least for the complexes **1**, **3**, and **5**. In the case of magnetic data obtained for complexes **2**, **4**, and **6**, there is a positive slope of the χ_Μ_ *vs. T* curve that eventually can be rationalized by the Van Vleck temperature-independent term α_TIM_. However, the low-temperature part of the susceptibility shows a drop of the effective magnetic moment (or the χ_M_*T* product function), which is a fingerprint of the non-uniform Bolztmann population of the *J*-multiplet energy levels split by the crystal field. Such a splitting term can be described by a set of Stevens operators in the form.

(2) $$\hat{H}=\underset{k=2,4,6}{\sum }\overset{+k}{\underset{q=-k}{\sum }}B_{k}^{q}{\hat{O}}_{k}^{q}\left(\hat{S_{z}},{\hat{S}}_{\pm }\right)$$

where $B_{k}^{q}$ are the interaction constants. Their number is too high in order to bracket them reliably based upon the powder susceptibility (magnetization) data. Therefore, we tried to fit the magnetic data with the $B_{2}^{0}$ and eventually the $B_{2}^{2}$ terms so that the Hamiltonian adopts the form (3) in the polar coordinate system.

(3) $${\hat{H}}_{\vartheta ,\varphi }=3B_{2}^{0}\left({\hat{J}}_{Z}^{2}-\frac{{\vec{J}}^{2}}{3}\right)+B_{2}^{2}\left({\hat{J}}_{x}^{2}-{\hat{J}}_{y}^{2}\right)+\mu_{B}Bg\left({\hat{J}}_{x}\sin\vartheta \cos\varphi \right)+{\hat{J}}_{y}\sin\vartheta \cos\varphi +{\hat{J}}_{z}\cos\vartheta$$

Its eigenvalues were calculated for 120 grids over one hemisphere, and the corresponding partition function, magnetization, and susceptibility were averaged. The results obtained are presented in the Supplementary Information, see Figures S2–S7.

## 3. Experimental Section

All chemicals were purchased from commercial sources (Sigma-Aldrich Co., Fluka Co., St. Louis, MO, USA) and were used as received without any further purification. The purity and composition of the prepared complexes were confirmed by means of elemental analysis, thermal analysis, electrospray-ionization mass spectrometry (ESI–MS), and FT-IR spectroscopy, and single crystal X-ray structure analysis.

Elemental analyses (CHN) were performed on a Flash 2000 CHNO-S Analyser (Thermo Scientific, Waltham, MS, USA).

The thermogravimetric analysis and differential thermal analysis (TG/DTA) were performed using the Exstar 6000 thermal analyzer (Seiko Instruments, Chiba, Japan) by heating the samples up to 1000 °C at the rate of 5 °C·min^-1^ in a dynamic air atmosphere (50 mL·min^−1^). Powder X-ray diffraction (XRD) data were obtained using a MiniFlex 600 diffractometer (Rigaku, Tokyo, Japan), with Cu *K*_α1,2_, in the range of 2–30 °2θ, with the scan speed of 2 °/min.

FT-IR spectra were recorded on a Nexus 670 FT-IR spectrometer (ThermoNicolet, Waltham, MS, USA) using KBr pellets (400–4000 cm^−1^) and the Nujol technique in the Far-IR spectral region (150–600 cm^−1^). The reported FT-IR signal intensities were defined as w = weak, m = medium, and s = strong.

Mass spectra of the water/methanol solutions (1/1, *v/v*) of the complexes were obtained by an LCQ Fleet ion trap mass spectrometer (Thermo Scientific, Waltham, MS, USA) in the negative ionization mode using the electrospray ionization (ESI–) technique. The thermal dependence of magnetic susceptibility measurements of the compounds were performed on an MPMS XL-7 SQUID magnetometer (Quantum Design, San Diego, CA, USA) in the temperature range of 300–1.8 K with an external field set to 0.1 T. The diamagnetic corrections were made by using the *Pascal* constants method by means of the MaTra2 software [36].

The visible photoluminescence of the prepared complexes was studied by steady-state photoluminescence spectroscopy using QuantaMaster 40 Spectrofluorometer (Photon Technology International, Inc., Birmingham, NJ, USA). The Schott UG1 band pass filter was placed between the excitation monochromator and the sample to cut off the stray light.

X-ray diffraction data were collected with a Rigaku HighFlux HomeLab™ universal dual wavelength (Mo–Kα and C–Kα) single crystal diffractometer at 120(2) K, while the Mo-Kα radiation (λ = 0.71075 Ǻ) was used to obtain the diffraction data. The diffractometer was equipped with the Eulerian 3 circle goniometer and the Rigaku Saturn724 + (2 × 2 bin mode) detector. Data reduction and correction of the absorption effect were performed using the XDS (http://xds.mpimf-heidelberg.mpg.de/) software package [37]. The structure was solved by direct methods using SHELXS-97 (http://shelx.uni-ac.gwdg.de/SHELX/) and refined on *F*^2^ using the full-matrix least-squares procedure (SHELXL-97) [38]. Non-hydrogen atoms were refined anisotropically and H-atoms were found from difference Fourier maps and refined using a riding model in most cases, while the O–H distances were treated using DFIX instructions. The molecular graphics as well as additional structural calculations were drawn and interpreted using Mercury, ver. 3.0 (http://www.ccdc.cam.ac.uk/Solutions/CSDSystem/Pages/Mercury.aspx) [39].

## 4. Conclusions

A series of six anionic heavy lanthanide(III) bis(*N*-salicylideneglycinato) complexes of the composition K[Ln(salgly)_2_(H_2_O)_2_]∙H_2_O (**1**–**6**), where Ln stands for Gd, Tb, Dy, Ho, Er and Tm, was prepared. The complexes were thoroughly characterized, including single crystal X-ray analysis of complex **2**. The results of temperature-dependence of magnetic susceptibility showed that the complexes behave as paramagnets obeying the Curie law with an added molecular field term. In the case of complexes **2**–**5**, the precision of data fitting was influenced by crystal field effects. Thus, these complexes could be magnetically interesting as such, or as the reactants to form the 3*d*-4*f* systems, which could show the properties of molecular magnets. The analysis of photoluminescence data measured in the solid phase and in water solutions uncovered the blocking mechanism of the solvent on the energy transfer between the Schiff base ligands and the lanthanide central atom. This feature might be interesting for the production of selective solid-phase sensors.

## Supplementary Materials

CCDC 1054730 contains the supplementary crystallographic data for complex **2**. These data can be obtained free of charge via http://www.ccdc.cam.ac.uk/conts/retrieving.html, or from the Cambridge Crystallographic Data Centre, 12 Union Road, Cambridge CB2 1EZ, UK; Fax: +44-1223-336-033; or E-Mail: deposit@ccdc.cam.ac.uk.

The results of the X-ray powder diffraction analyses of complexes **1**–**6** are given in the Supplementary Information (see Figure S1). The magnetic data fitting using a set of Stevens operators is presented in Supplementary Information (see Figures S2–S7).

Supplementary materials can be found at http://www.mdpi.com/1422-0067/16/05/9520/s1.
