# Supplementary material for: Structural, Magnetic and Luminescent Properties of Lanthanide Complexes with N-Salicylideneglycine
Source: Int J Mol Sci. 2015 Apr 28;16(5):9520–39. doi: 10.3390/ijms16059520 (PMC4463602; doi:10.3390/ijms16059520)
Supplement: Supplementary file 1 [file ijms-16-09520-s001.pdf]

Supplementary Information

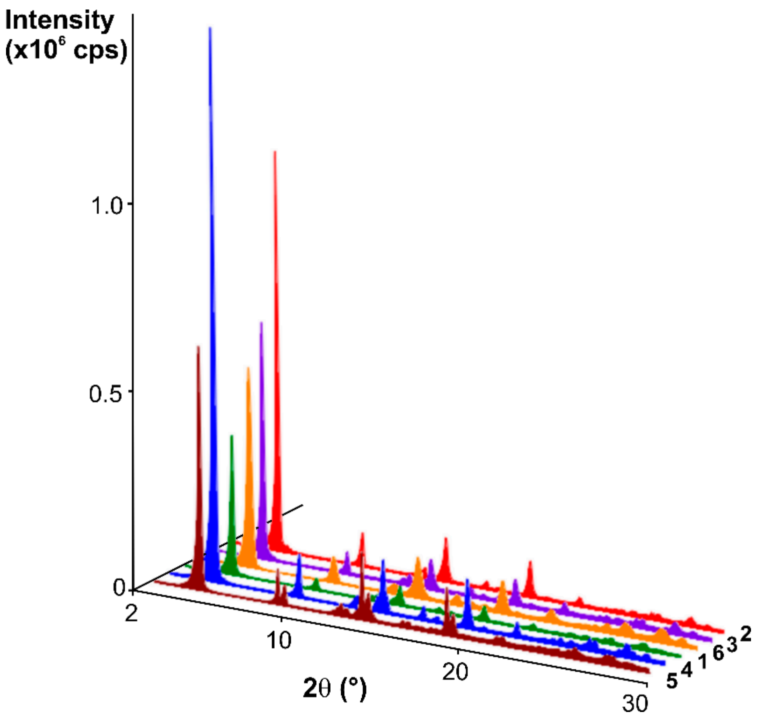

Figure S1. A comparison of powder XRD data of complexes **1**–**6**, showing similarity in their XRD patterns.

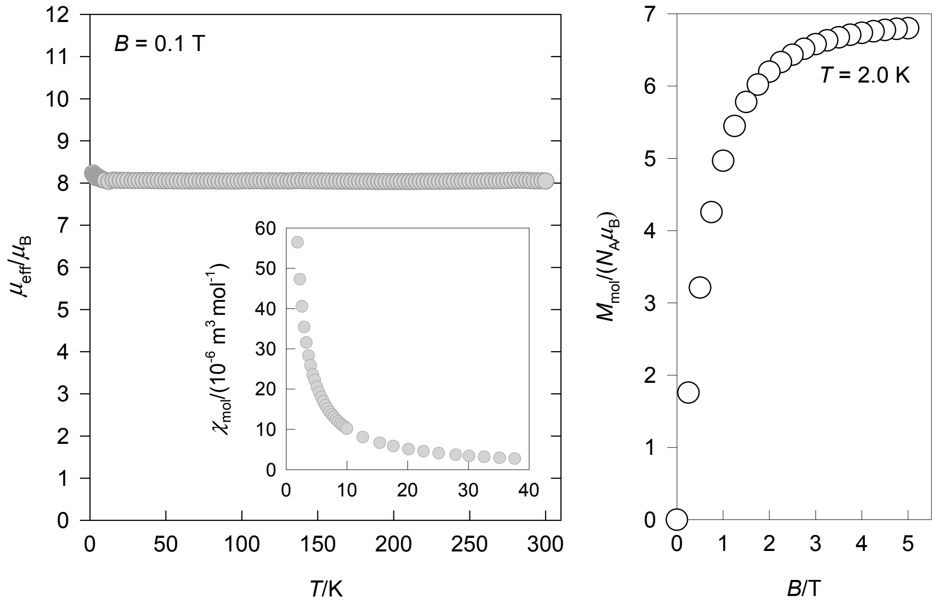

Figure S2. Magnetic data for **1** (Gd). (**Left**) temperature dependence of the effective magnetic moment per formula unit; (**Right**) field dependence of the magnetization. Circles—experimental data.

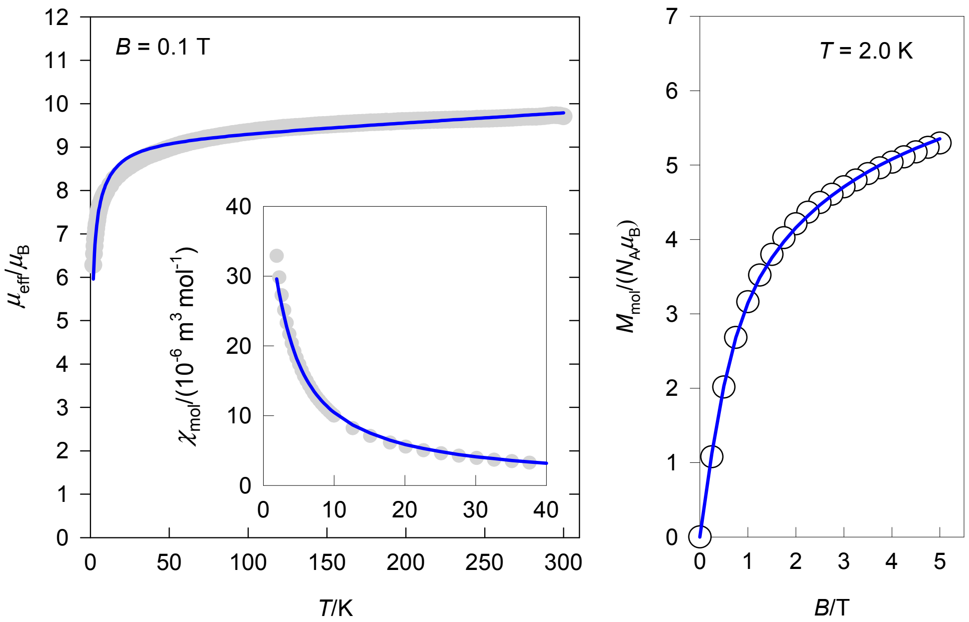

Figure S3. Magnetic data for **2** (Tb). (**Left**) temperature dependence of the effective magnetic moment per formula unit; (**Right**) field dependence of the magnetization. Circles—experimental data, solid lines—with $g = 1.403$, $3B_2^0/hc = 6.39$ cm^{-1}, $B_2^2/hc = 0.20$ cm^{-1}, $\alpha_{TIM} = 70.0 \times 10^{-9}$ m^3·mol^{-1}, $zj/hc = -0.009$ cm^{-1}; $R(\chi) = 0.050$, $R(M) = 0.0089$.

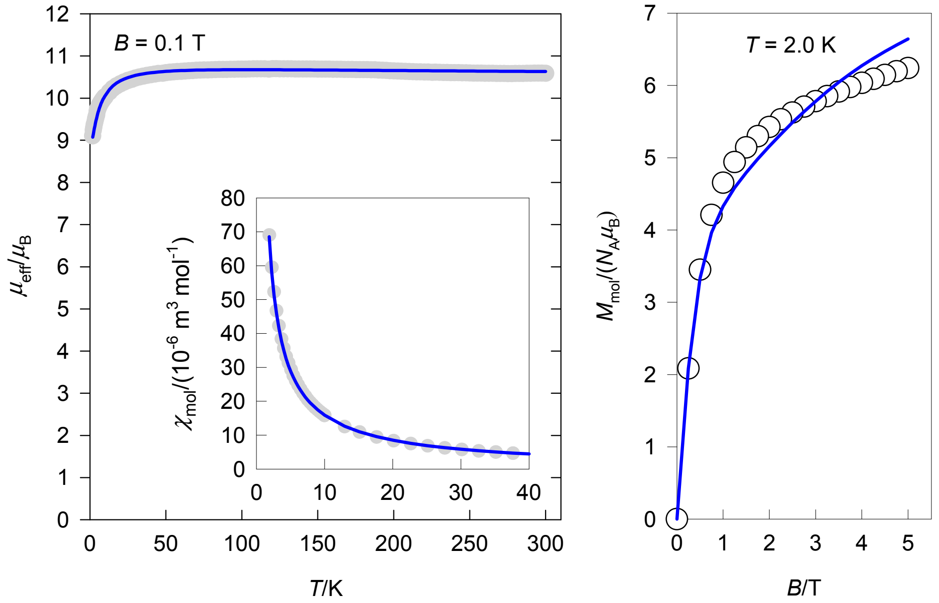

Figure S4. Magnetic data for **3** (Dy). (**Left**) temperature dependence of the effective magnetic moment per formula unit; (**Right**) field dependence of the magnetization. Circles—experimental data, solid lines—fitted with $g = 1.332$, $3B_2^0/hc = 3.26$ cm^{-1}, $B_2^2/hc = 0.15$ cm^{-1}, $\alpha_{TIM} = -4.9 \times 10^{-9}$ m^3·mol^{-1}, zj/hc~0; $R(\chi) = 0.016$, $R(M) = 0.046$.

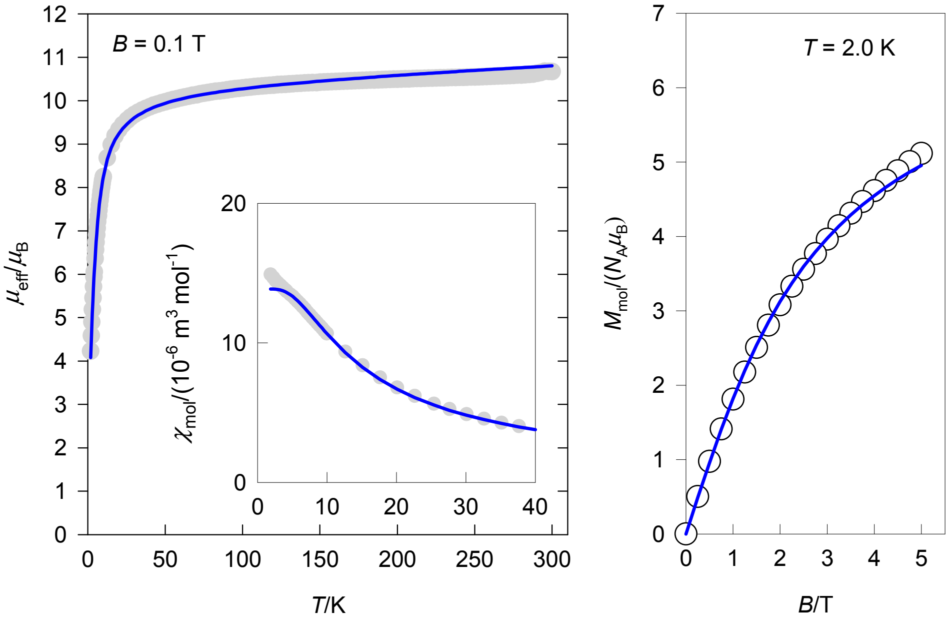

Figure S5. Magnetic data for **4** (Ho). (**Left**) temperature dependence of the effective magnetic moment per formula unit; (**Right**) field dependence of the magnetization. Circles—experimental data, solid lines—fitted with g = 1.195, $3B_2^0/hc$ = 17.69 cm^{-1}, B_2^2/hc = 0.15 cm^{-1}, α_{TIM} = 65.0 × 10^{-9} m^3·mol^{-1}, zj/hc = 0.0029 cm^{-1}; $R(\chi)$ = 0.024, $R(M)$ = 0.018.

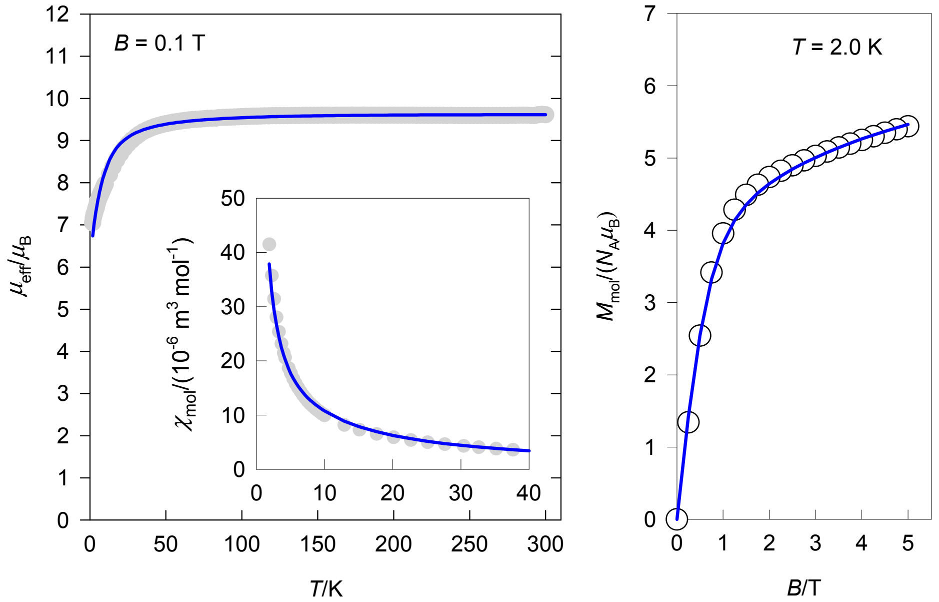

Figure S6. Magnetic data for **5** (Er). (**Left**) temperature dependence of the effective magnetic moment per formula unit; (**Right**) field dependence of the magnetization. Circles—experimental data, solid lines—fitted with g = 1.194, $3B_2^0/hc$ = 12.7 cm^{-1}, zj/hc = −0.012 cm^{-1}; $R(\chi)$ = 0.059, $R(M)$ = 0.016.

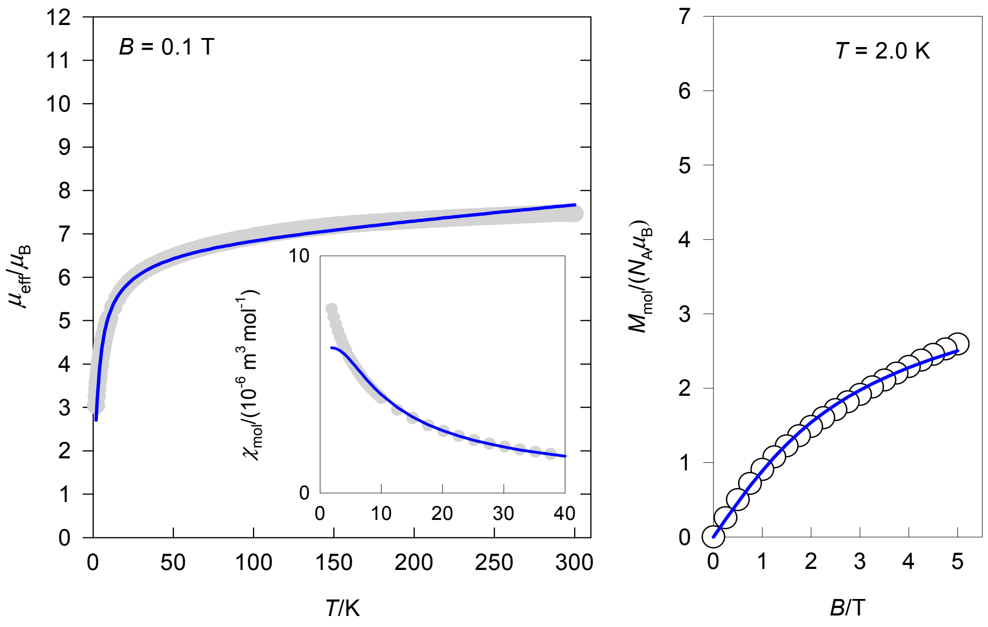

Figure S7. Magnetic data for **6** (Tm). (**Left**) temperature dependence of the effective magnetic moment per formula unit; (**Right**) field dependence of the magnetization. Circles—experimental data, solid lines—fitted with g = 1.016, $3B_2^0/hc$ = 19.87 cm^{-1}, B_2^2/hc = 0.63 cm^{-1}, α_{TIM} = 83.4 × 10^{-9} m^3·mol^{-1}, zj/hc = −0.047 cm^{-1}; $R(\chi)$ = 0.089, $R(M)$ = 0.027.
